# Supplementary material for: Identification and functional analysis of non-coding regulatory small RNA FenSr3 in Bacillus amyloliquefaciens LPB-18
Source: PeerJ. 2023 May 15;11:e15236. doi: 10.7717/peerj.15236 (PMC10194069; doi:10.7717/peerj.15236)
Supplement: Supplemental Information 4 [file peerj-11-15236-s004.zip › KO/CK-vs-T1_map/map01120.html]

KEGG PATHWAY: Microbial metabolism in diverse environments - Reference pathway


|  |  |
| --- | --- |
| **Microbial metabolism in diverse environments - Reference pathway** |  |

[
Pathway menu
| Organism menu
| Pathway entry
|

Hide module list

| User data mapping
|

Image (png) file

]

|  |  |  |
| --- | --- | --- |
| Reference pathway | 100% 70% 35% | 图片下载 |

- **Category title**

  

- **KEGG module**

- Energy metabolism
  - Carbon fixation
    - M00165 Reductive pentose phosphate cycle (Calvin cycle)- M00166 Reductive pentose phosphate cycle, ribulose-5P => glyceraldehyde-3P
      - M00167 Reductive pentose phosphate cycle, glyceraldehyde-3P => ribulose-5P
      - M00168 CAM (Crassulacean acid metabolism), dark
      - M00169 CAM (Crassulacean acid metabolism), light
      - M00172 C4-dicarboxylic acid cycle, NADP - malic enzyme type
      - M00171 C4-dicarboxylic acid cycle, NAD - malic enzyme type
      - M00170 C4-dicarboxylic acid cycle, phosphoenolpyruvate carboxykinase type
      - M00173 Reductive citrate cycle (Arnon-Buchanan cycle)
      - M00376 3-Hydroxypropionate bi-cycle
      - M00375 Hydroxypropionate-hydroxybutylate cycle
      - M00374 Dicarboxylate-hydroxybutyrate cycle
      - M00377 Reductive acetyl-CoA pathway (Wood-Ljungdahl pathway)
      - M00579 Phosphate acetyltransferase-acetate kinase pathway, acetyl-CoA => acetate
      - M00620 Incomplete reductive citrate cycle, acetyl-CoA => oxoglutarate
  - Nitrogen metabolism
    - M00175 Nitrogen fixation, nitrogen => ammonia- M00531 Assimilatory nitrate reduction, nitrate => ammonia
      - M00530 Dissimilatory nitrate reduction, nitrate => ammonia
      - M00529 Denitrification, nitrate => nitrogen
      - M00528 Nitrification, ammonia => nitrite
      - M00804 Complete nitrification, comammox, ammonia => nitrite => nitrate
  - Methane metabolism
    - M00567 Methanogenesis, CO2 => methane- M00357 Methanogenesis, acetate => methane
      - M00356 Methanogenesis, methanol => methane
      - M00563 Methanogenesis, methylamine/dimethylamine/trimethylamine => methane
      - M00358 Coenzyme M biosynthesis
      - M00608 2-Oxocarboxylic acid chain extension, 2-oxoglutarate => 2-oxoadipate => 2-oxopimelate => 2-oxosuberate
      - M00174 Methane oxidation, methanotroph, methane => formaldehyde
      - M00346 Formaldehyde assimilation, serine pathway
      - M00345 Formaldehyde assimilation, ribulose monophosphate pathway
      - M00344 Formaldehyde assimilation, xylulose monophosphate pathway
      - M00378 F420 biosynthesis
      - M00422 Acetyl-CoA pathway, CO2 => acetyl-CoA
  - Sulfur metabolism
    - M00176 Assimilatory sulfate reduction, sulfate => H2S- M00596 Dissimilatory sulfate reduction, sulfate => H2S
      - M00595 Thiosulfate oxidation by SOX complex, thiosulfate => sulfate
- Carbohydrate and lipid metabolism
  - Central carbohydrate metabolism
    - M00004 Pentose phosphate pathway (Pentose phosphate cycle)- M00006 Pentose phosphate pathway, oxidative phase, glucose 6P => ribulose 5P
      - M00007 Pentose phosphate pathway, non-oxidative phase, fructose 6P => ribose 5P
      - M00580 Pentose phosphate pathway, archaea, fructose 6P => ribose 5P
      - M00008 Entner-Doudoroff pathway, glucose-6P => glyceraldehyde-3P + pyruvate
      - M00308 Semi-phosphorylative Entner-Doudoroff pathway, gluconate => glycerate-3P
      - M00309 Non-phosphorylative Entner-Doudoroff pathway, gluconate/galactonate => glycerate
  - Other carbohydrate metabolism
    - M00373 Ethylmalonyl pathway- M00740 Methylaspartate cycle
- Nucleotide and amino acid metabolism
  - Aromatic amino acid metabolism
    - M00533 Homoprotocatechuate degradation, homoprotocatechuate => 2-oxohept-3-enedioate- M00545 Trans-cinnamate degradation, trans-cinnamate => acetyl-CoA
  - Other amino acid metabolism
    - M00033 Ectoine biosynthesis, aspartate => ectoine
  - Cofactor and vitamin biosynthesis
    - M00622 Nicotinate degradation, nicotinate => fumarate- M00550 Ascorbate degradation, ascorbate => D-xylulose-5P
      - M00836 Coenzyme F430 biosynthesis, sirohydrochlorin => coenzyme F430
- Secondary metabolism
  - Aromatics degradation
    - M00538 Toluene degradation, toluene => benzoate- M00537 Xylene degradation, xylene => methylbenzoate
      - M00419 Cymene degradation, p-cymene => p-cumate
      - M00547 Benzene/toluene degradation, benzene => catechol / toluene => 3-methylcatechol
      - M00548 Benzene degradation, benzene => catechol
      - M00551 Benzoate degradation, benzoate => catechol / methylbenzoate => methylcatechol
      - M00637 Anthranilate degradation, anthranilate => catechol
      - M00568 Catechol ortho-cleavage, catechol => 3-oxoadipate
      - M00569 Catechol meta-cleavage, catechol => acetyl-CoA / 4-methylcatechol => propanoyl-CoA
      - M00539 Cumate degradation, p-cumate => 2-oxopent-4-enoate + 2-methylpropanoate
      - M00543 Biphenyl degradation, biphenyl => 2-oxopent-4-enoate + benzoate
      - M00544 Carbazole degradation, carbazole => 2-oxopent-4-enoate + anthranilate
      - M00418 Toluene degradation, anaerobic, toluene => benzoyl-CoA
      - M00541 Benzoyl-CoA degradation, benzoyl-CoA => 3-hydroxypimeloyl-CoA
      - M00540 Benzoate degradation, cyclohexanecarboxylic acid =>pimeloyl-CoA
      - M00534 Naphthalene degradation, naphthalene => salicylate
      - M00638 Salicylate degradation, salicylate => gentisate
      - M00624 Terephthalate degradation, terephthalate => 3,4-dihydroxybenzoate
      - M00623 Phthalate degradation, phthalate => protocatechuate
      - M00636 Phthalate degradation, phthalate => protocatechuate
      - M00810 Nicotine degradation, pyridine pathway, nicotine => succinate semialdehyde
      - M00811 Nicotine degradation, pyrrolidine pathway, nicotine => succinate semialdehyde
